# Micro RNA Molecules as Modulators of Treatment Resistance, Immune Checkpoints Controllers and Sensitive Biomarkers in Glioblastoma Multiforme

**DOI:** 10.3390/ijms21041507

**Published:** 2020-02-22

**Authors:** Marek Mazurek, Jakub Litak, Piotr Kamieniak, Ida Osuchowska, Ryszard Maciejewski, Jacek Roliński, Wiesława Grajkowska, Cezary Grochowski

**Affiliations:** 1 Department of Neurosurgery and Pediatric Neurosurgery, Medical University of Lublin, Jaczewskiego 8, 20-954 Lublin, Poland; marekmazurek@hotmail.com (M.M.); jakub.litak@gmail.com (J.L.); pkamieniak@poczta.onet.pl (P.K.); 2 Department of Immunology, Medical University of Lublin, Chodźki 4a, 20-093 Lublin, Poland; jacek.rolinski@gmail.com; 3 Department of Anatomy, Medical University of Lublin, Jaczewskiego 4, 20-090 Lublin, Poland; i.m.osuchowska@gmail.com (I.O.); maciejewski.r@gmail.com (R.M.); 4 Department of Oncopathology and Biostructure, „Pomnik-Centrum Zdrowia Dziecka” Institute, Al. Dzieci Polskich 20, 04-730 Warsaw, Poland; w.grajkowska@ipczd.pl; 5 Laboratory of Virtual Man, Department of Anatomy, Medical University of Lublin, Jaczewskiego 4, 20-090 Lublin, Poland

**Keywords:** micro RNA, glioblastoma multiforme, immune checkpoints, glioma, tumor

## Abstract

Based on genome sequencing, it is estimated that over 90% of genes stored in human genetic material are transcribed, but only 3% of them contain the information needed for the production of body proteins. This group also includes micro RNAs representing about 1%–3% of the human genome. Recent studies confirmed the hypothesis that targeting molecules called Immune Checkpoint (IC) open new opportunities to take control over glioblastoma multiforme (GBM). Detection of markers that indicate the presence of the cancer occupies a very important place in modern oncology. This function can be performed by both the cancer cells themselves as well as their components and other substances detected in the patients’ bodies. Efforts have been made for many years to find a suitable marker useful in the diagnosis and monitoring of gliomas, including glioblastoma.

## 1. Introduction

Only in the United States about 10,000 new cases of high grade glioma are detected annually [1]. Despite the relatively low prevalence due to high aggressiveness, the tumor is responsible for about 4% of cancer-related deaths [2]. Glioblastoma multiforme belongs to a group of primary nervous system tumors, characterized by high aggressiveness in terms of the histological as well as clinical aspect. It was described as IV grade glioma according to the World Health Organization (WHO) classification. GBM represents 10%–18% of all intracranial tumors and 50%–60% of all astrocytoma tumors. The annual occurrence of GMB is 5/100,000 persons, mostly diagnosed in the 5 th and 6 th decade. There are two forms of GBM-primary and secondary. The first one occurs most often in people over 55 years of age. It is characterized by rapid clinical manifestation and de novo occurrence, which already are typical for GBM. In the secondary form, usually diagnosed in people under 45 years of age, the tumor develops on the basis of low grade.

The 5-year survival rate is found to be below 20% in patients suffering from glioblastoma multiforme, while the median survival time after treatment reaches ca. 11–24 months [3].

Based on genome sequencing, it is estimated that over 90% of genes stored in human genetic material are transcribed, but only 3% of them contain the information necessary for the production of body proteins [4]. This group also includes micro RNAs (miRNA) representing about 1%–3% of the human genome [5].

About 40% of the micro RNA genes are located in the introns and exons of other genes, which may be associated with their basic function, i.e., regulation of the expression of other genes [6,7,8].

Furthermore, each micro RNA can control up to over 100 target points and each of which can be regulated by different miRNAs. The result of this is the creation of a complicated system of key dependences in the regulation of basic functions of the organism [5,9,10].

The first papers describing the presence of micro RNA were published in 1990s [11]. The role of miRNA was pointed out in many medical conditions such as kidney disease, diabetes, neurodegenerative disorder, arthritis, as well as cancer [12]. MiRNAs are also involved in studies on reproductive function and infertility [13,14].

In the 2000s, the first papers describing the importance of micro RNA in the pathophysiology of brain tumors, including glioblastoma, began to appear [15,16,17]. Since then, many authors have been analyzing the effects of these particles on various aspects of glioblastoma development. Numerous observations have shown the disturbances between the level of expression of micro RNA compared to normal brain tissue [18,19,20,21,22,23]. Analysis of the literature indicates that in the case of glioblastoma, the level of micro RNA (253 up-regulated *vs*. 95 down-regulated) is overexpressed more often [11]. Among the particles with increased expression, the authors most often mentioned: miR10b, miR17-92, miR21, miR26a, miR92 miR221/222, miR335, miR451, and miR486 while the low levels were often presented by: miR7, miR34a, miR106a, miR124, miR128, miR129, miR137, miR139, miR-181a, miR181b, miR218, miR323 and miR328, and miR342-3p [16,17,19,23,24,25,26,27,28,29,30,31,32,33,34]. The importance of many of these micro RNA in the pathogenesis of cancer is still unknown, but in recent years intensive research has been carried out to clarify this issue.

## 2. Immune Checkpoints vs. MirRNA

Recent studies confirmed the hypothesis that targeting molecules called Immune Checkpoint (IC) open new opportunities to take control over GBM. The cytotoxic T-lymphocyte–associated antigen 4 (CTLA-4) was the first particle with immunomodulatory properties, blocked with Ipilimumab (humanized antibody) in patients with metastatic melanoma [35]. CTLA-4 inhibits proper T cells co-stimulatory signaling by binding CD 86 and CD 80 on antigen-presenting cells. In patients suffering from GBM, there is a negative correlation with the expression of molecules on the CD8+ and CD4+ cells and the final outcome of the disease [36,37]. Programmed death ligand 1 (PD-L1) as a member of IC family plays a crucial role in immune modulation and immune adaptation of glioma cells. Activation of PD-1 receptor on T-cells results in significant reduction of its cytotoxicity and proliferation. Further signaling increases tumor evasion and leads to immune escape. Over-expression of PD-L1 is preceded by the stimulation of multiple receptors such as epidermal growth factor receptor (EGFR), interferon receptors (IFNAR, IFNGR) and toll like receptors (TLRs). Down-signaling of self-activated glioma cells takes place via several pathways such as: phosphatase and tensin homolog deleted on chromosome ten/the mammalian target of rapamycin (PTEN/mTOR), signal transducer and activator of transcription 3/mitogen-activated protein kinase/extracellular signal-regulated kinases (STAT3/MEK/ERK) or Janus-activated kinases/signal transducers and activators of transcription (JAK/STAT), which promote PD-1L mRNA transcription, translation and presentation on the surface of cell [38,39]. Micro RNA molecules have an ability to affect described processes. Wang et al. presented an analysis of various (49) miRNA particles regulating PD-L1 and PD-1 interplay in oncologic patients. The immunological variability of GBM harmonizes with IDH 1/2 mutations. Heiland et al. confirmed that PD-L1 is up-regulated in IDH1/2 wildtype contrary to the IDH1/2 mutated cells which hypermethylate the PD-L1 promoter region, resulting in lower levels of PD-L1 expression. Those findings could explain various responses to inhibition of PD-L1 in patients qualified in recruiting trials and point at molecules interacting with ICs on the epigenetic level such as miRNAs. Moreover, miRNAs interfere with PD-1/ PDL1 and CTLA-4 pathways. Particular molecules interplay with precise IC: miR-28 (PD-1), miR-34a (PD-L1), mir-124 (PD-1), mir-138 (PD-1 and CTLA-4), miR-138-5p (PD-L1), mir-155 (CTLA4), miR-200 (PD-L1) miR-424 (PD-L1), and miR-513, (PD-L1) [36,37]. miRNAs could also interact with IC indirectly. Some of them induce IFN gamma secretion and control transcription, creating complicated connections. Studies over MiR-34a revealed an anti-tumor effect on glioma cells by modifying epidermal growth factor receptor (EGFR) and PD-L1 expression. MiR-34a silences FKBP51 (cochaperone molecule of PD-L1), interrupting the maturation of PD-L1. This action reduces resistance to treatment induced by the PD-L1/PD-1 axis. The expression of miR-34a correlates with better outcomes [40,41,42]. Epigenetic studies performed by Chen et al. concluded that the miR-200 particle represents a reciprocal connection with the expression of PD-L1 [43]. Wei et al. discovered the same relation between Pd-L1 and miR-138-5p. According to his study, MiR-138 targets also PD-1 and CTLA-4, and suppresses expression on the surface of CD4+ T cells. Studies carried out on mice models demonstrate tumor mass regression and significant elongation of median survival time. The regression of the tumor maintained after miR-138 intake discontinuation [44]. Moreover, miR-124 inhibits signal transducer and the activator of transcription 3 (STAT3) signaling, leading to down-regulation of immunosuppressive particles such as PD-1. 

**Figure 1:**
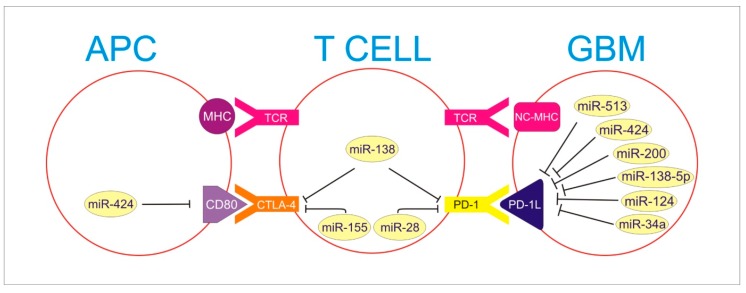
APC, T cell and GBM interplay in activation of immune checkpoints resulting in immune escape of Glioma. The figure presents miRNA molecules developing an inhibitory influence on the presented axis. PD-1 and CTLA-4 on T cell interact with miR-138, inhibiting both. MiR-155 inhibits CTLA-4, and miR-28 inhibits PD-1 as well.

The expression of MiR-124 is limited in high-grade gliomas and in all types of malignant tumors. miR-124 affects glioma cancer stem cells (gCSC) modulated with immunosuppressive properties. Systemic management enhances anticancer properties and modulates glioma microenvironment to protect immunoescape [45,46,47]. Up-regulation of miR-155 promotes the development of regional inflammation, which results in tumor invasion via CTLA-4 activation [48]. Mir-28 control replication processes reduce PD-1 expression attaching to 3‘UTR. This causes T cells depletion by modulating cell cycles [49]. miR-424 and miR 513 inhibit PD-1L formation in glioma. miR 424 additionally inhibits CD 80 molecule on antigen-presenting cell (APCs), resulting indirect inhibition of CTLA-4 signaling [46,47] (Figure 1). 

PD-1L in GBM is blocked by miR-513, miR 424, miR-200, miR-138-5p, miR-124, and miR 34a. Moreover, miR-424 blocks CD80 particle on APC, which is necessary to activate CTLA-4 receptor on T cell.

MHC—Major Histocompatibility Complex; NC-MHC—Non Classical Major Histocompatibility Complex; TCR—T Cell Receptor; CTLA—4-Cytotoxic T Cell Antigen 4; PD-1—Programmed Death Receptor-1; CD80—Cluster of Differentation 80; PD-1L—Programmed Death-Ligand 1.

Moreover, IFN-gamma down-regulates MiR-513 and its restrictive activity on PD-1L expression in GBM cells. Despite promising results on animal models, further studies are required to explain and clarify the complicated interplay between miRNAs and ICs.

## 3. Biomarkers

The detection of markers that indicate the presence of cancer plays a very important role in modern oncology. This property can be presented by both the cancer cell itself as well as their components and other substances detected in the patients bodies [50,51]. Efforts have been made for many years to find a suitable marker useful in the diagnosis and monitoring of gliomas, including glioblastoma [52,53]. However, to this day, it has not been possible to identify a substance that would fulfill this role properly [54]. In the case of GMB, monitoring the level of biochemical markers would be particularly important because of the difficulties linked to imaging diagnostics. Under the influence of the applied treatment, the tumor cells absorb larger amounts of administered contrast, thus leading to an adulterated radiosensitive image acquisition, significantly impeding the control of therapy [55]. For this reason, a great contribution is required to discover new markers such as micro RNAs.

The importance of miRNA playing a role as a marker of the different pathologies has already been suggested in Alzheimer’s disease as well as other cancers such as: papillary thyroid carcinoma, breast cancer, metastasis in gastric cancer, Hodgkin lymphoma, and pancreatic ductal adenocarcinoma [56,57,58,59,60,61]. One of the first studies on the importance of miRNA in the diagnosis of gliomas was conducted on astrocytoma samples. Zhi et al. indicated seven miRNA particles, in which levels were significantly different from healthy brain tissue [62]. Likewise, many other studies were conducted, including high-grade glioma [63]. Observations by Chen et al. indicated another seven examples of micro RNA with potential use as markers. The group consisted of: miR7, miR15b, miR21, miR124a, miR129, miR139, and miR218 [64]. In a large meta-analysis carried out by Sirnivasan et al., the role of 10 miRNAs that could be used as a predictor of the disease progression in patients with GBM was highlighted. Moreover, a great number of them were associated with a worse course of the disease, while others indicated a good prognosis. The first group included: miR31, miR146b, miR148a, miR193a, miR200b, and miR221 [65]. A similar relationship in the case of miR146b was also observed in the case of squamous cell lung cancer and papillary thyroid carcinoma [66]. Interestingly, some researchers emphasize the importance of this molecule in suppressing metastatic changes [67,68]. Similar observations have been made regarding breast cancer and miR31 [69]. Likewise, the role of miR221 in tumors pathogenesis, including GBM, has been confirmed in studies by other authors. Particular attention was paid to the participation of p27, p53, PTEN, and p27Kip1 particles in this mechanism [70,71,72,73,74,75,76]. In the case of miR193a, correlations with adverse prognosis were observed regarding melanoma [77]. Sirnivasan et al. included miR17-5p, miR20a and miR106a as substances that could potentially have protective properties [67]. A similar relationship to miR106a has been found in other studies on glioblastomas and colorectal cancer [64,78]. On the other hand, in the case of T-cell leukemia, the oncogenic potential of this particle has been demonstrated [79]. Similar conclusions regarding the positive prognostic significance of miR17 and miR20a (together included in the miR17-92 cluster) in gliomas were suggested in studies of the goals of these particles (cyclin D1, E2F1) [80,81,82]. Moreover, in observations carried out on various types of cancers, it was found that they can perform both oncogenic and suppressor functions in the cell [83,84,85,86,87,88,89]. Many researchers also pointed out other miRNAs in which presence may be associated with better prognosis and longer survival of patients with glioma and glioblastoma. Thanks to this, these can potentially be used as biomarkers of the course of the disease. This group includes, among others (Table 1): miR29c, miR101, miR107, miR144-3p, mir181d, miR203, miR205, and miR328 [30,90,91,92,93,94]. 

In another large analysis conducted by Qiu et al. on 480 GMB samples, it was noted that patients with longer survival rates showed high levels of miR130a and miR326, as well as low levels of miR155, miR210, miR323, and miR329. Moreover, in the case of miR155, miR210, miR130a, and miR326, this was also associated with progression-free survival (PFS) [95]. These data remain consistent with the observations of Schiesser et al. They confirmed that low levels of miR155 and miR210 reflect worse prognosis. In their analysis, 29 miRNA regions differing in methylation emphasized that only miR155 was a prognostic marker independent of other patient characteristics such as MGMT methylation, IDH1/IDH2 mutation and histology [96].

The correlation of the presence of some miRNAs with patients life expectancy is often associated with the degree of histological staging of the tumor. In the work carried out by Regazzo et al., it has been shown that testing the level of expression of some miRNAs (miR125b, miR497) can provide information on whether the examined tumor belongs to high-grade glioma or low grade [97]. Similar conclusions were made by Wang et al. stating that the level of miR342-3p in the serum of patients with gliomas is lowered, while the level of this decrease is different for tumors with grade II, III and IV [24]. The existence of similar correlations has been demonstrated by many other researchers, including: miR19, miR137, miR144-3p, and miR182 [98,99,100,101]. It was also found that the relationship between the stage of tumor histology is positive for miR19 and miR182, and negative for le7e, miR145 and miR181a [102,103]. This would provide valuable guidance on the characteristics of the tumor before surgery and histopathological examination of the slice, giving the chance for better planning of the procedure. Observing the level of markers in the form of miRNA could thus capture the moment of progression of low-grade gliomas in high-grade glioma. Malkorn et al. conducted research on WHO grade II gliomas that progressed to GBM (WHO IV). They indicated a number of markers helpful in determining the moment of histological progression. They included: miR9, miR15a, miR16, miR17, miR19a, miR20a, miR21, miR25, miR28, miR130b, and miR140; miR210 (elevated level); and miR184 and miR328 (reduced level) [104]. In their study, Wang et al. also found that the levels of miR21, miR128 and miR342-3p change under the influence of anti-cancer treatment (returns to levels characteristic of healthy tissue) [24]. In the case of miR128, similar trends were also reported in other studies [93]. This indicates the possibility of using miRNA as markers of treatment effectiveness.

## 4. Resistance to Treatment

Treatment of glioblastoma, due to the dynamics of its development, is a big challenge for modern medicine. Currently, the commonly accepted standard of treatment includes a combination of cytoreductive surgery, chemotherapy and radiotherapy. Not every patient will respond equally to the implemented treatment. It is caused, among others, by chemoresistance associated with: repair mechanisms, apoptosis, epigenetic regulations, and different accumulations of drugs in cells [105]. Micro RNA can play an important role in regulating these phenomena. In recent years, numerous observations have been made in this field [106,107,108,109,110,111,112].

Temozolomide (TMZ) is a cytostatic agent commonly used in the therapy of patients with GBM. It has been shown to be highly effective in reducing tumor growth, having a positive effect on patient survival [113,114]. Also in this case, there were observations describing the cell resistance to the drug. Micro RNA can also participate in this mechanism [115]. The cytostatic properties of TMZ are associated with alkylation of the guanine bases in DNA, consequently leading to impaired DNA replication and cell death [116,117,118]. However, in the case of neoplasm, some cells have developed mechanisms to avoid this scenario. An example of this is the O6-methylguanine-DNA methyl-transferase (MGMT). It is an enzyme involved in DNA repair by removing alkylated groups from the O6 guanine position, preventing mispairing in the process of DNA replication. The effect is a reversal of the cytostatic and lack of effectiveness of the therapy [119,120]. Despite the existence of other mechanisms conditioning chemoresistance, such as DYNC2H1 (DHC2) activity, MGMT is considered to be a critical regulator of temozolomide insensitivity [121]. 

Inactivation of O6-methylguanine-DNA methyl-transferase would allow blocking repair processes, thereby increasing the effectiveness of temozolomide [122]. This can be achieved by epigenetic mechanisms such as methylation. The first studies regarding silencing of MGMT in gliomas date back to 2005 [115]. In observations carried out by Hegi et al., the relationship between MGMT methylation determined by methylation-specific polymerase-chain-reaction and the survival of glioblastoma patients was examined. The results indicated that the methylation of the O6-methylguanine-DNA methyl-transferase promoter predicted a positive response to treatment regardless of other factors [115]. For this reason, many research facilities are trying to find an effective method to weaken MGMT activity. 

D’Atri et al. suggested that this effect could be achieved using combined TMZ therapy with cisplatin [123]. Unfortunately, further observations indicated a relatively low effectiveness of this combination in patients with glioblastoma [124]. Similar conclusions were received in the case of radiation therapy [125]. Many authors have tried to explain the mechanism of this type of resistance. Interestingly, Chen et al. and Guo et al. suggested that a certain group of micro RNAs could be good markers of patients’ poor response to cisplatin treatment. This concerned, among others, miR873 and Let7b [126,127,128].

**Figure 2:**
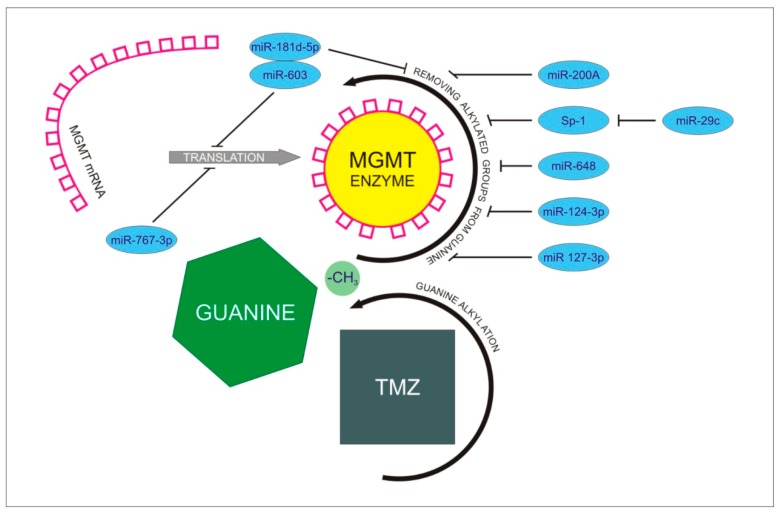
Presentation of MGMT-dependent mechanism leading to the development of resistance to TMZ treatment. TMZ administration intensifies alkylation processes concerning guanine nucleotides in glioblastoma cells. MGMT mRNA is translated to active MGMT enzyme, resulting in intensive dealkylation of guanine. Reverse effect of guanine alkylation leads to promotion of treatment resistance. MiR 767-3p and miR-181d-5p/miR 603 complex inhibit MGMT translation to prevent dealkylation. MiR-200A, miR-648, miR-124-3p, and miR 127-3p inhibit removing alkylated groups from guanine performed by MGMT enzyme. MiR-29c inhibits indirect dealkylation of guanine through Sp-1 protein.

Recent observations show that micro RNA can condition the chemosensitivity of cancer cells also by acting on MGMT. In their work, Berthois et al. noted the inverse correlation between miR200a levels and O6-methylguanine methyltransferase (MGMT) expression. They also showed that over-expression of miR200a causes sensitization of glioblastoma cells to TMZ and that it can serve as a good marker of tumor chemosensitivity [129]. In another work by Kushwah et al., the role of micro RNA in increasing the chemosensitivity of the tumor was also described. In this case, the observations concerned miR603. The authors pointed out that miR-603 binds to the MGMT 3’UTR resulting in a decrease in O6-methylguanine-DNA methyl-transferase activity. Interestingly, they also emphasized the role of miR603 and miR181d cooperation in the regulation of MGMT activity [130]. This coincided with their earlier work, which highlighted the role of miR181d in the mechanism of glioblastoma chemosensitivity [131] (Figure 2). 

### Sp-1- Specificity Protein-1

Other authors also pointed to the role of the aforementioned miR29c in regulating MGMT activity (Table 2). However, in this case, the micro RNA acts on the enzyme through specificity protein 1 (Sp1). It contributes to inhibition of O6-methylguanine-DNA methyl-transferase and an increase in the sensitivity of glioblastoma cells to TMZ [90].

On the other hand, research by Slaby et al. conducted on 22 patients with primary glioblastoma suggested that there was no correlation between the survival rate of chemoradiotherapy-treated patients and MGMT methylation status. However, the authors found that miR181b and miR181c levels may serve as a marker of a positive response of glioblastoma patients to chemoradiotherapy with TMZ. Patients who had good sensitivity to the applied treatment showed lower levels of these micro RNAs. The lack of association with MGMT activity suggests the involvement of other mechanisms in regulating cell response to cytostatic [132]. Also, Ujifuku et al. in their research on acquired resistance to TMZ in glioblastoma multiforme cells, indicated the independence of this parameter from the methylation status of O6-methylguanine-DNA methyl-transferase [133]. Auger et al. also received similar results in previous observations of glioma cell lines [134]. This indicates the need for further research to fully understand the differences in glioblastoma sensitivity to treatment.

One of the mechanisms that can affect the chemoresistance of cells independent of MGMT is apoptosis. Wang et al. showed that miR143 overexpression is associated with a greater sensitivity of gliomas to TMZ treatment. The authors stated that the reason for this phenomenon is the reduction of RAS viral oncogene homolog (N-RAS) neuroblastoma expression, which is the direct target of micro RNA [135]. The relationship between proapoptotic mechanisms (regulation of Bcl genes) and the sensitivity of glioblastoma cells to temozolomide has also been shown in studies by Kouri et al. and Li et al. In these cases, miR182 and miR139 were involved in the regulation of apoptosis [136]. In the research of Shi et al., the opposite correlation was described for miR21 activity. Overexpression of this micro RNA was associated with inhibition of the sensitivity of GBM cells to TMZ [137]. An analogous effect of miR21 was also seen in the observations of other authors, also in the case of resistance to other drugs such as doxorubicin, paclitaxel, carmustine, VM-26, and sunitinib [138,139,140,141,142,143].

The sensitivity of tumor cells to cytostatics is also influenced by the activity of metadherin (MTDH), also known as astrocyte elevated gene-1 protein (AEG-1). It is a protein involved in the process of angiogenesis [144]. It has been shown that it also plays a large role in the induction of chemoresistance in cancer cells [145]. For gliomas, lowering its level increases the sensitivity of cells to temozolomide [146]. Wu et al. research showed that this occurs with the increase in expression of miR136, whose direct target is MTDH [147]. This indicates the potential role of miR136 as a marker of glioma cell sensitivity to cytostatics.

In recent years, the potential role of several further micro RNAs in the induction of chemoresistance has been discovered. In the aforementioned work of Ujifuku et al., the authors pointed to miRNAs whose titters were particularly increased in resistant cells. They included miR10a, miR195 and miR455-3p. At the same time, they noted that reducing miR195 levels contributed most to increased tumor sensitivity to temozolomide therapy. Other researchers also mentioned in this group: miR17-5p, miR101, miR155, miR193a-5p, miR203, miR204, miR221, miR222, and miR328. This effect was determined by the impact on a number of metabolic pathways as well as cellular transporters as in the case of miR328 [148,149,150,151,152,153,154,155].

Furthermore, micro RNA can condition resistance not only to cytostatics but also to radiation therapy. This happens, among others, by affecting ATM (ATM serine/threonine kinase). It is a protein involved in the mechanisms of apoptosis, DNA repair and cell cycle arrest [156]. Tribius et al. studies have shown that it also plays an important role in regulating radiosensitivity in primary glioblastoma. The authors noted that the sensitivity of cells to radiation therapy is inversely correlated with the ATM level [157]. In another study by Ng et al., it has been shown that miR100 can negatively affect the ATM level, thereby affecting the radiosensitivity of glioma cells [158].

## 5. Conclusions

MicroRNA has great variety of applications both in the diagnostic processes as a potential tumor biomarker as well as in the treatment of glioblastoma multiforme. The immunological checkpoints encoded by microRNA can be a target of therapy, supporting commonly used methods such as radiochemotherapy and classical surgery.

## Figures and Tables

**Table 1:** Level of expression of particular mRNA molecules in Glioblastoma Multiforme.

Increased	Increased 2-10x	Increased More 10x	Fold	Decreased	Decreased 2-10x	Decreased More 10x
miR26a miR221/222 miR335 miR451 miR486 miR501-3p	miR15b miR17-5p miR23a miR21 miR25 miR104 miR106b miR124 miR148a miR182 miR183 miR188 miR199a miR199b miR199s miR200cN miR210N miR224 miR368 miR373	miR10a miR10b miR96	iR30a-3p miR143 miR186 miR324-3p miR337 miR355	miR106a miR181a miR181b miR448 miR490 miR876-3p	miR29cN miR31 miR95 miR107 miR122a miR149 miR154 miR190 miR219 miR221 miR299 miR321 miR323 miR328 miR331 miR340 miR342 miR370	miR33 miR105 miR124a miR124b miR128a miR128b miR129 miR132 miR133a miR133b miR137 miR139 miR153 miR154 miR184 miR203 miR218 miR330 miR326 miR338

**Table 2:** Presentation of miRNA, responsible for the resistance of glioblastoma cells to treatment.

MiRNA.	Target
miR-29c	Through specificity protein 1 (Sp1), it contributes to inhibition of O6-methylguanine-DNA methyl-transferase
miR-143	the reduction of RAS viral oncogene homolog (N-RAS) neuroblastoma expression
miR-182	Repression of Bcl2-like12 (Bcl2L12), c-Met, and hypoxia-inducible factor 2α (HIF2A)
miR-139	targeting MDA-9/syntenin
miR-200a	Direct transcriptional repressor of zipper containing kinase (ZAK)
miR-648	Represses luciferase activity
miR-124-3p	Suppresses the expression of endothelia receptor type B
miR-127-3p	Target the tumor-suppressor gene SEPT7
miR-767-3p	Degradation of the MGMT mRNA
miR-603	inhibiting Wnt inhibitory factor 1 (WIF1) and β-catenin-interacting protein 1 (CTNNBIP1)
miR-181d-5p	Degradation of the MGMT mRNA
